# Sex-Specific Muscular Maturation Responses Following Prenatal Exposure to Methylation-Related Micronutrients in Pigs

**DOI:** 10.3390/nu9010074

**Published:** 2017-01-18

**Authors:** Michael Oster, Nares Trakooljul, Henry Reyer, Annette Zeyner, Eduard Muráni, Siriluck Ponsuksili, Klaus Wimmers

**Affiliations:** 1 Leibniz Institute for Farm Animal Biology (FBN), Institute for Genome Biology, Wilhelm-Stahl-Allee 2, 18196 Dummerstorf, Germany; oster@fbn-dummerstorf.de (M.O.); trakooljul@fbn-dummerstorf.de (N.T.); reyer@fbn-dummerstorf.de (H.R.); murani@fbn-dummerstorf.de (E.M.); ponsuksili@fbn-dummerstorf.de (S.P.); 2 Martin-Luther-University Halle-Wittenberg, Department of Animal Nutrition, Theodor-Lieser-Str. 11, 06120 Halle (Saale), Germany; annette.zeyner@landw.uni-halle.de

**Keywords:** fetal programming, maternal diet, methyl donors, myogenesis, one-carbon cycle, pigs

## Abstract

Supplementation of micronutrients involved in DNA methylation, particularly during pregnancy, is recommended because of its impacts on human health, but further evidence is needed regarding the effects of over-supplementation and differences between sexes. Here, a porcine model was used to assess effects of maternal supplementation with one-carbon-cycle compounds during prenatal and postnatal stages on offspring muscle development. Sows received either a standard diet (CON) or a standard diet supplemented with folate, B6, B12, methionine, choline, and zinc (MET) throughout gestation. Myogenesis-, growth-, and nutrient utilization-related transcript expression was assessed using quantitative PCR. Organismal phenotype and gene expression effects differed significantly between males and females. Male MET-offspring showed increased fetal weight during late pregnancy but decreased live weight postnatally, with compensatory transcriptional responses comprising myogenic key drivers (*Pax7*, *MyoD1*, *myogenin*). In contrast, female weights were unaffected by diet, and mRNA abundances corresponded to a phenotype of cellular reorganization via *FABP3*, *FABP4*, *SPP1* and Insulin-like Growth Factor-signaling. These findings in an animal model suggest that supplementation during pregnancy with methylation-related micronutrients can promote sex-specific myogenic maturation processes related to organismal growth and muscle metabolism. The usage of maternal dietary supplements should be more carefully considered regarding its ability to promote fetal and postnatal health.

## 1. Introduction

Micronutrients involved in processes related to DNA methylation (e.g., folate, vitamins B6 and B12, methionine, choline, zinc) are commonly provided as supplements during pregnancy to benefit offspring development. However, an excessive maternal intake of methylation-related micronutrients acts as a nutritional insult, inducing hypermethylation at epi-labile loci and culminating in phenotypic [1,2] and metabolic variation [3,4]. In particular, some maternal dietary methylation-related supplementation regimens have been shown to adversely affect skeletal muscle tissue development in murine fetuses [5,6,7]. Since rodents require a higher relative dose of such micronutrients than humans or pigs [8,9], porcine models have been used to study paternal or maternal dietary supplementation with methylation-related micronutrients [8,10,11]. In pigs, high maternal intake of these micronutrients is associated with increased fetal mass during late gestation, i.e., during the period when secondary muscle fibers are formed and hyperplasia occurs [12]. Extracts obtained from fetal skeletal muscle implicate insulin-like growth factor (IGF)-related compounds in the observed weight differences [8]. Indeed, in vitro exposure to the methylation-related micronutrient folate affects muscle myoblast differentiation via the IGF downstream target Akt and transcription factors of the myogenic lineage, such as MyoD and myogenin [13]. Furthermore, the phenotypic and transcriptomic effects of dietary supplementation with methylation-related micronutrients have been described to be sex-specific [14,15,16,17]. Thus, important gaps exist in our knowledge regarding the beneficial effects of dietary methylation-related micronutrient supplementation.

Shifts in body weight, muscle fiber distribution, myogenesis, and nutrient utilization could be detrimental for health [18]. The goal of this study was to determine whether a maternal diet enriched with methylation-related micronutrients affects transcripts involved in myogenesis, growth, and nutrient utilization. Potential sex effects were considered. The composition of muscle by fiber type can be assessed by detecting gene expression patterns of Myosin heavy chain isoforms (MyHC) [19]. In porcine fetuses and juvenile pigs, expression of key factors was analyzed in *M. longissimus dorsi* to approximate proliferative and metabolic features, at the transcriptional level, of cells originating from the myogenic lineage.

## 2. Materials and Methods

### 2.1. Animals, Diets, Sample Collection

**Figure 1:**
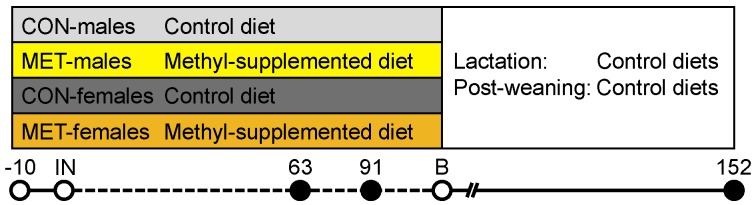
Experimental design. Piétrain gilts were fed either a CON or a MET diet from 10 days before insemination (IN) until tissue sampling at 63 dpc, 91 dpc, and 152 dpn, respectively, when *M. longissimus dorsi* (*n* = 40 per stage) was collected. IN—insemination; B—birth; dpc—days post conception; dpn—days post natum.

The experimental setup was in accordance with the Animal Protection legislation of the federal state and of the country of Mecklenburg-Western Pomerania, Schwerin, Germany. Animal care and tissue collection procedures were approved by the Scientific Committee of the Leibniz-FBN (70.1.2.03.201). The animal experiment was performed as described previously [8]. Briefly, Piétrain gilts were randomly assigned to receive either a standard diet (CON) or a standard diet supplemented with one-carbon-cycle substrates and associated cofactors (MET), starting from 10 days before artificial insemination throughout pregnancy (Figure 1). Specifically, methionine, choline, folic acid, vitamin B6, vitamin B12, and zinc were supplemented (Table 1). The doses of the altered micronutrients match about 80% of their known tolerable upper intake levels. For dietary vitamin B12 levels, no overdosing is known [20]. Hence, the chosen supplementation was adapted to high doses used in human studies [21,22] to ensure maximal uptake of vitamin B12. Gilts were individually reared in cages on flat decks in environmentally controlled rooms. Access to pelleted feed has been restricted to 2.8 kg/day. The body weight and feed intake during gestation were not significantly different between gilts fed the CON or MET diets. The number of fetuses/offspring per sow did not differ significantly due to the maternal diets.

At 63 dpc and 91 dpc, a subset of gilts were exsanguinated and uteri were quickly removed and dissected (*n* = 3 per stage and diet). Fetal muscle tissue (*M. longissimus dorsi*) was immediately collected, frozen in liquid nitrogen, and stored at $-80{\;}^{\circ }C$ until further analyses (63 dpc: *n* = 40; 91 dpc: *n* = 40). Another subset of gilts (*n* = 3 per diet) was kept until delivering live-born progeny (114 dpc). During lactation, sows received a standard lactation diet. Male piglets were castrated at 4 dpn by authorized qualified personnel in approved user’s establishments. Post-weaning (28 dpn, week 4), the progeny was fed control diets ad libitum. The progeny’s muscle tissue (*M. longissimus dorsi*) was collected at 152.3±2.6 dpn, frozen in liquid nitrogen, and stored at $-80{\;}^{\circ }C$ until further analyses (*n* = 40). In total, the study comprises 120 individual samples (*n* = 9–11 per sex per stage per dietary group). Notably, pigs attain sexual and reproductive maturity not before six months of age [23,24].

### 2.2. Phenotype Data Analyses

Weights and carcass characteristics were analyzed via variance analysis (version 9.4., SAS Institute, Cary, NC, USA), and effects represented by diet, sex, and gilt were included. A repeated measurement statement was applied to compute postnatal live weights. The carcass characteristics were corrected for live weight. Differences were considered significant at p≤0.05.

### 2.3. RNA Isolation and cDNA Synthesis

In total, 120 muscle tissue samples (*n* = 40 per stage) were used to isolate total RNA using Tri-Reagent per manufacturer’s directions (Sigma-Aldrich, Taufkirchen, Germany). Quantification and purification were performed as previously described [8]. All RNA samples were stored at $-80{\;}^{\circ }C$ until downstream analyses were performed. Samples were checked for contamination by genomic DNA. First-strand cDNA was synthesized from 2 μg of total RNA using random primers and oligo d(T) 13VN in the presence of Superscript III reverse transcriptase (Invitrogen, Karlsruhe, Germany). The cDNA samples were purified using QIAquick PCR purification kit (Qiagen, Hilden, Germany), eluted in 20 μL Aqua dest. and stored at $-20{\;}^{\circ }C$ until further analyses.

### 2.4. Primer Sequences and Primer Validation

A number of transcripts associated with myogenesis, growth, myosin heavy chains, lipid metabolism, and energy metabolism were analyzed. mRNA sequences of both target and reference genes were obtained from the National Center for Biotechnology Information (NCBI) Gene database (NCBI, Bethesda, MD, USA). Primer sequences are listed in Table S2. The specificity of primer sequences was checked using tissue-specific test samples. Transcript levels of selected target and reference genes were quantified by qPCR performed on a LightCycler 480 system (Roche, Mannheim, Germany) according to the manufacturer’s instructions. Briefly, reactions were performed in a final volume of 10 μL using 5.0 μL of LightCycler 480 SYBR Green I Master (Roche), 0.5 μL (10 μM) of each primer, 2 μL (40 ng) cDNA, and 2.0 μL of Aqua dest. The temperature profiles were comprised of an initial denaturation step at $95{\;}^{\circ }C$ for 10 min followed by 40 cycles consisting of denaturation at $95{\;}^{\circ }C$ for 15 s, annealing at $60{\;}^{\circ }C$ for 10 s and extension/fluorescence acquisition at $72{\;}^{\circ }C$ for 15 s. Amplified products were subjected to melting curve analyses and gel electrophoresis to verify the absence of non-specific products.

### 2.5. Microfluidic High-Throughput qPCR

Selected target and reference genes were analyzed in duplicate on a BioMarkTM HD system (Fluidigm, San Francisco, CA, USA) following the manufacturer’s instructions (*n* = 40 per stage; balanced by diet and sex). In brief, a pre-amplification step was performed in a final volume of 5 μL using 2.5 μL of TaqMan PreAmp Master Mix (Applied Biosystems, Waltham, MA, USA), 0.75 μL of Aqua dest., 0.5 μL (500 nM) of pooled primer mixture (comprising aliquots of all primers set to be included), and 1.25 μL purified cDNA. The temperature profile was comprised of an initial denaturation step at $95{\;}^{\circ }C$ for 10 min followed by 10 cycles consisting of $95{\;}^{\circ }C$ for 15 s and $60{\;}^{\circ }C$ for 4 min. Subsequently, a clean-up was performed by adding 0.2 μL exonuclease I reaction buffer, 0.4 μL exonuclease I (20 U/μL), and 1.4 μL of Aqua dest. The temperature profile consisted of $37{\;}^{\circ }C$ for 30 min and $80{\;}^{\circ }C$ for 15 min. Furthermore, the pre-amplified and cleaned-up cDNA was diluted (1:10) using Tris-EDTA (Ethylenediaminetetraacetic acid) buffer (10mM Tris/HCl; 1.0 mM EDTA).

Using the 96.96 dynamic array, PCRs were performed in a final volume of 10 μL. Specifically, 5 μL sample premix (2.5 μL SsoFast EvaGreen Supermix (Bio-Rad Laboratories, Munich, Germany) with low 6-carboxy-X-rhodamine (ROX), 0.25 μL DNA Binding Dye Sample Loading Reagent, 2.25 μL pre-amplified and diluted cDNA) and 5 μL assay premix (2.5 μL Assay Loading Reagent, 2.25 μL DNA Suspension Buffer, 0.25 μL primer mixture) were prepared and transferred to the primed array. Subsequently, the final PCR mix was formed due to the integrated fluidic circuits and physical loading protocols finally generating 22,080 data points (92 transcripts measured in duplicate in 40 samples at 3 time points). The final primer concentration per individually performed PCR was 500 nM. The temperature profile was comprised of an initial denaturation step at $95{\;}^{\circ }C$ for 60 s followed by 30 cycles consisting of $96{\;}^{\circ }C$ for 5 s and $60{\;}^{\circ }C$ for 20 s. Raw data (quantification cycle) was obtained using BioMarkTM Data Collection software (version 4.1.3, Fluidigm, San Francisco, CA, USA).

### 2.6. Transcript Data Preprocessing and Analysis

Expression data were compared to quality control criteria and predicted outliers were removed (melting curve of amplified products, lower and upper bound of quantification cycle) as proposed by the manufacturer (Real-time PCR analysis software, version 4.1.3, Fluidigm, San Francisco, CA, USA). To account for variation in RNA input and efficiency of reverse transcription, values were normalized by geometric averaging of reference genes as described previously [25,26]. Transcriptional alterations due to diet and sex were analyzed stage-specifically via variance analysis (SAS, version 9.4., SAS Institute, Cary, NC, USA), and effects represented by diet, sex, and gilt confounded with diet were included. To account for multiple testing, *p*-values were converted to a set of *q*-values [27]. Unless specified, differences were considered significant at p≤0.05 and q≤0.30. Fold changes displaying differences in mRNA abundances were calculated from least square means (positive FC: CON < MET; negative FC: CON > MET).

To model the diversity of muscle fiber types, data of transcripts associated with myosin heavy chain isoforms (*MYH1*, *MYH2*, *MYH3*, *MYH4*, *MYH7*) were used to calculate a principal component analysis. The first two dimensions were used to plot diet- and sex- dependent distribution (eigenvalue > 1 for each principal component).

### 2.7. Validation of Microfluidic High-Throughput qPCRs

To verify microfluidic high-throughput qPCRs, selected target (*FABP4*, *HSD11B1*, *MYH4*, *SPP1*) and reference genes (*RPL32*, *RPL10*) were quantified by qPCR performed on a LightCycler 480 system (Roche, Mannheim, Germany) as described above (*n* = 40 per stage). Data was factorial normalized and analyzed as described above (SAS). The level of significance was set at p≤0.05. Correlation of normalized expression values was calculated by Spearman’s Rho.

## 3. Results

The aim of the current study was to investigate whether prenatal exposure to methylation-related micronutrients (“methyl-supplemented”, or MET, versus standard dosing, or CON) affects muscle development of the offspring, and its implication for organismal growth. Responsiveness to the dietary challenge was analyzed in male and female pigs via weight recordings and expression profiling at different ontogenetic stages.

### 3.1. Maternal Supplementation with Methylation-Related Micronutrients Affected Fetal Weight and Live Weight in Males

**Figure 2:**
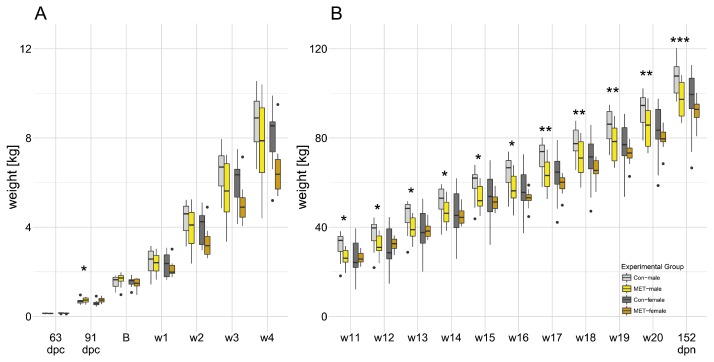
Box plots representing fetal weights and live weights in progeny exposed to maternal CON and MET diets. Data recorded (**A**) at fetal stages display individual experiments. Data recorded from (**A**) birth to week 4 (28 dpn), (**B**) from week 11 (77 dpn) to week 20 (140 dpn), and at 152 dpn represents a time course. Fetal weight was increased in MET-males at 91 dpc. Live weight was lowered in MET-males from week 11 (77 dpn) until 152 dpn. CON—Standard diet; MET—Standard diet supplemented with methylating micronutrients; CON-males: **light gray**; CON-females: **dark gray**; MET-males: **yellow**; MET-females: **orange**; * p≤0.05; ** p≤0.01; *** p≤0.001; dpc—days post conception; dpn—days post natum.

Weights at fetal stages (63 dpc; 91 dpc) and live weights (birth weight until 152 dpn) are displayed in Figure 2. Litter size was unaffected between CON and MET sows. At 63 dpc, fetal weight did not differ by diet between groups of males (CON-males: 127.8±5.6 g; MET-males: 141.5±5.6 g; *p* = 0.094) or females (CON-females: 151.0±5.4 g; MET-females: 142.4±4.9 g; *p* = 0.243). However, at 91 dpc, fetal weight was significantly higher in methyl-supplemented males (CON-males: 677.8±26.6 g; MET-males: 790.4±34.5 g; *p* = 0.015) and tended to be higher in methyl-supplemented females (CON-females: 656.8±27.3 g; MET-females: 728.6±23.8 g; *p* = 0.056) compared with sex-matched controls. Birth weight was unaffected by maternal diet in both males and females. Postnatally, MET-males had a significantly lower live weight than CON-males at 77 dpn (week 11), but females exhibited no difference in live weights by diet. Accordingly, at 152 dpn, live weight was significantly different by diet between males (CON-males: 105.0±1.6 kg; MET-males: 95.1±1.7 kg; *p* < 0.001) but not different between females (CON-females: 94.2±1.7 kg; MET-females: 92.7±2.1 kg; *p* = 0.564).

### 3.2. Increased Percent Lean Mass in MET-Males

Postnatal body characteristics are displayed in Table S1. Specifically, percent lean mass was higher in MET-males compared to CON-males (CON-males: 59.02% ±0.40%; MET-males: 60.57% ±0.53%; *p* = 0.030), but similar between females (CON-females: 61.67% ±0.41%; MET-females: 61.62% ±0.52%; *p* = 0.935). Body characteristics related to back fat, pH, and meat color were not affected by diet or sex.

### 3.3. Gene Expression Pattern Specific for Maternal Diet and Sex

In MET-males (63 dpc), transcripts of *Akt1*, *FLT4*, and *VEGFB* were more abundant compared to CON-males (p≤0.05 but corresponding *q*-value of 0.68) (Table 2 and Table S2). At 91 dpc, transcripts associated with growth (*PDGFA*, *VEGFC*), myogenesis (*MYF6*), and lipid metabolism (*PPARD*) were less abundant in MET-males compared to CON-males (corresponding *q*-values < 0.30). At 152 dpn, MET-males showed greater mRNA abundances of *MyoD1*, *Pax7*, and *myogenin* (corresponding *q*-values < 0.30).

In MET-females, transcripts associated with fiber formation (*FBXO32*, *MSTN*, *MYF5*), lipid metabolism (*PPARA*), and energy metabolism (*PFKM*, *PPARGC1A*, *PRKAA2*) were more abundant compared to CON-females at 63 dpc. At 91 dpc, transcripts related to IGF signaling (*Akt1*, GSK3β, *PIK3CD*, *PIK3CG*), myogenic control (*FST*), growth (*FLT1*, *FLT4*, *KDR*), lipid metabolism (*FABP3*, *FABP4*, *PPARA*), and energy metabolism (*GALK1*, *GLUT1*, *GLUT4*, *PC*) were more abundant in MET-females compared to CON-females. At 152 dpn, differences for *SPP1* and *MET* reached p≤0.05; however, the corresponding *q*-value was 0.99.

### 3.4. Myosin Heavy Chain Isoforms

**Figure 3:**
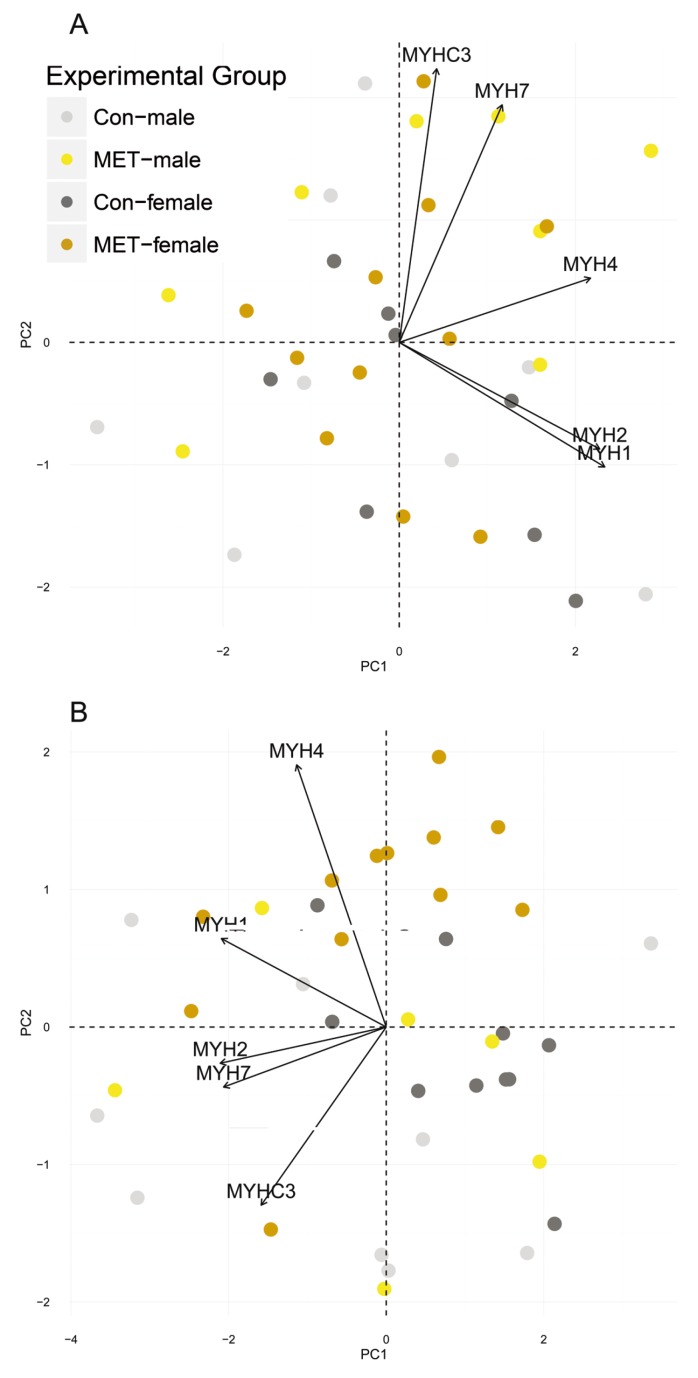

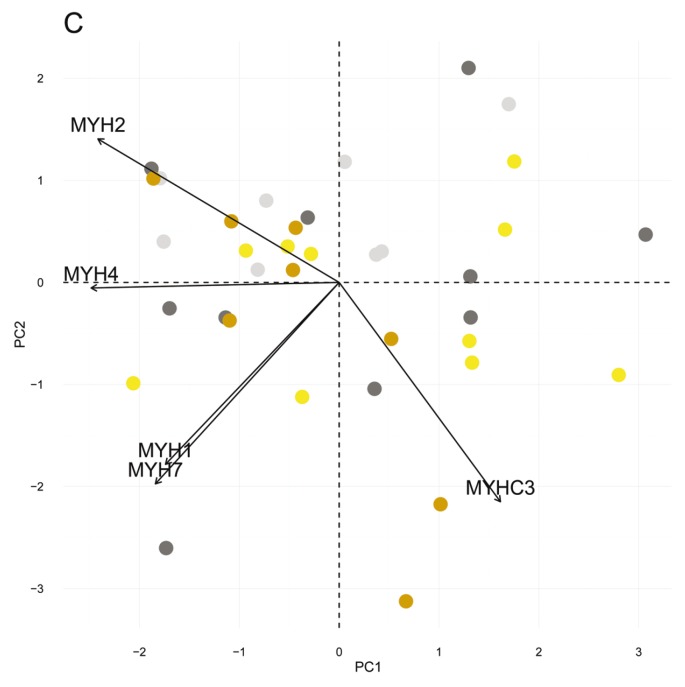
Principal component analysis-biplot of individual pigs and myosin heavy chain isoforms. Expression data of *MYH1*, *MYH2*, *MYH3*, *MYH4*, and *MYH7* was used to describe differences among individual pigs affiliated with CON-males (**light gray**), CON-females (**dark gray**), MET-males (**yellow)**, and MET-females (**orange**) at (**A**) 63 dpc, (**B**) 91 dpc, and (**C**) 152 dpn, respectively. CON—Standard diet; MET—Standard diet supplemented with methylating micronutrients; dpc—days post conception; dpn—days post natum.

At 63 dpc and 152 dpn, mRNA abundances of myosin heavy chain isoforms (*MYH1*, *MYH2*, *MYH3*, *MYH4*, *MYH7*) were unaffected by diet or sex. At 91 dpc, *MYH2* and *MYH4* were upregulated in MET-females (Table 2, Tables S2 and S3). Accordingly, a principle component analysis (PCA) calculated for expression values of myosin heavy chain isoforms revealed that samples were unsystematically distributed at 63 dpc and 152 dpn (Figure 3). At 91 dpc, the cluster of MET-females might follow the shifts of the variance components represented by both *MYH2* and *MYH4*.

### 3.5. Verification of qPCR Results

The qPCR results obtained via Fluidigm and LightCycler 480 systems were correlated to verify differences in mRNA abundance of selected transcripts, specifically for *FABP4*, *HSD11B1*, *MYH4*, and *SPP1* (Table S3). Significant correlations between expression values ranged between 0.67 and 1.00, which suggest reliable results. The fold change (FC) was reproducible on both systems. Although results of *MYH4* measured via high-throughput qPCR did not differ (*p* = 0.08, Table S3), expression values retrieved from the LightCycler 480 system were increased in MET-females at 91 dpc.

## 4. Discussion

Skeletal muscle is a dynamic tissue that adapts to external stimuli through transcriptional, biochemical, and morphological variations. This study focused on the time during which primary and secondary fiber hyperplasia are completed, measuring growth performance and mRNA abundances of key factors involved in myogenesis, proliferation, and energy utilization. Male pigs exposed to methylation-related micronutrients exhibited higher fetal weight during late pregnancy but lower live weight during the postnatal period. Effects on organismal weight following excessive intrauterine exposure to methylation-related micronutrients have been reported in studies on mixed sexes, but findings were inconsistent, apparently due to either single or combined effects of such nutrients [4,28,29,30]. Thus, our study supports previous work indicating that methylation-related micronutrients can affect offspring growth (“methyl-supplemented”, or MET, versus standard dosing, or CON).

The IGF system may have a prominent role in mediating the observed weight effects [8,31,32]. In our study, phenotypic variations were reflected by compensatory transcriptional variations: the prenatal expression of growth-associated transcripts (*MYH6*, *PDGFA*, *VEGFC*) was lower, but the postnatal expression of myogenic key drivers (*Pax7*, *MyoD1*, *myogenin*) was higher following MET exposure. The emphasis on the expression of transcripts involved in driving myogenic precursor proliferation, such as satellite cells, might fit to the observation that MET-males exhibited greater lean mass percentage at 152 dpn. Indeed, postnatal muscle growth takes place primarily via myofiber hypertrophy [33]. However, it has been shown previously that muscle regulatory factors (MRFs) involving *Pax7*, *MyoD1*, and *myogenin* implied the occurrence of activated satellite cells [34], which differentiate to myoblasts, fuse to myotubes, and finally mature into myofibers [35,36,37]. Since the mitotically quiescent satellite cells are activated via trauma, extrinsic mechanical stretch, or growth factors, the data might suggest that the response to supplemented methylation-related micronutrients changed the program of myogenic differentiation. At 152 dpn, when animals were sampled, the line between adolescence and adulthood is fading; thus, it is conceivable that MET-exposed males exhibit prolonged growth and maturation processes by exploiting the high plasticity of satellite cells during the postnatal period.

In female pigs, fetal and postnatal live weights were unaffected by the dietary challenge. However, a previous study found that female offspring exhibited decreased weight following methylation-related micronutrient supplementation during pregnancy [38]. In our study, MET-exposed females exhibited transcriptional alterations in *FABP3* and *FABP4*, which are known to act as biomarkers for muscle fiber types and lipid accumulation and are implicated in adverse metabolic states [39,40]. Moreover, transcripts associated with IGF signaling were more abundant. Since members of the GLUT family orchestrate glucose uptake and are regulated by Akt, which is, in turn, phosphorylated by PI3K, the maternal supplementation with methylation-related micronutrients might act on myogenesis and muscle differentiation during fetal development [41,42]. Furthermore, the analyses revealed increased expression of *MYH2* and *MYH4*, for which the variance components derived from a PCA of myosin heavy chain isoforms might parallel a shift from CON-females to MET-females. Whether this indicates a cellular shift towards MyHC2a and MyHC2b fibers remains unclear since no fiber typing was performed. However, maternal diets varying in micro- and macronutrient supply during gestation have been shown to affect the formation of myofibers [43,44,45]. Postnatally, *SPP1* had greater abundance, which may highlight a dietary impact on myogenesis [46,47] since *SPP1* is known to be regulated by the muscle regulatory factors *MyoD1* and *MYF5* [48]. Increased expression levels of *SPP1* are implicated in pathophysiological states associated with obesity and macrophage recruitment, as reviewed elsewhere [49,50].

Taken together, males might have responded via systemic mechanisms culminating in growth alterations with compensatory transcriptional responses. However, female responses comprised cellular features, i.e., metabolic demands might have pushed females toward a status of cellular reorganization (by using glycolytic features) with an emphasis on muscle fibers and adipocytes. The results suggest that male and female animals favored different strategies to adapt to the dietary challenge. The data suggest that the maternal diet might impact myogenesis. An implication for organismal growth and muscle cell reorganization is conceivable, which might account for sex-specific maturation processes. Thus, supplementing methylation-related micronutrients like folate, B6, B12, methionine and choline during pregnancy at higher-than-recommended doses may alter organismal processes beyond preventing birth defects.

In humans, maternal supplementation with methylation-related micronutrients may benefit individuals exposed to prenatal dietary undersupply or even famine, which still burdens a considerable proportion of the global population [51]. However, our findings in a porcine model indicate that it might be useful to consider diversified nutritional recommendations by offspring sex. To maximize the therapeutic potential of methylation-related micronutrients, further investigations (e.g., histological or metabolic studies) are warranted because potential metabolic risks are of scientific interest and public health importance.

## 5. Conclusions

In summary, the study demonstrated that there are effects due to the supplementation of methylation-related micronutrients at the phenotypic and transcriptomic level. The study revealed sex-dependent strategies to adapt to a prenatal exposure to methylation-related micronutrients. Potentially, growth and maturation processes may be prolonged in MET-exposed males, whereas the programming of female muscle tissue might result in a cellular reorganization. Hence, known advantageous phenotypes mediated by methylation-related micronutrient intake during pregnancy should be further discussed with regard to their acute and persistent impact on muscle plasticity with a special emphasis on sexual dimorphism.

## Figures and Tables

**Table 1:** Dietary amount of altered micronutrients fed to gilts during gestation (per kg diet).

Methylating Micronutrient	Sow CON Diet	Sow MET Diet
Methionine, mg	2050	4700
Choline, mg	500	2230
Folic acid, mg	3	92.2
Vitamin B6, mg	3	1180
Vitamin B12, μg	31	5930
Zinc, mg	21.8	149

CON—Standard diet; MET—Standard diet supplemented with methylating micronutrients.

**Table 2:** Transcripts affected by diet and sex at prenatal and postnatal time points (excerpt from Table S2).

Gene	Males 63 dpc		Females 63 dpc		Males 91 dpc		Females 91 dpc		Males 150 dpn		Females 150 dpn	
	*p*	FC ^1^	*p*	FC ^1^	*p*	FC ^1^	*p*	FC ^1^	*p*	FC ^1^	*p*	FC ^1^
Akt1	0.026 *	+1.40	0.002	+1.51	ns	ns	0.046	+1.29	ns	ns	ns	ns
FABP3	ns	ns	ns	ns	ns	ns	0.001	+1.38	ns	ns	ns	ns
FABP4	ns	ns	ns	ns	ns	ns	0.031	+1.39	ns	ns	ns	ns
FBXO32	ns	ns	0.038	+1.33	ns	ns	ns	ns	ns	ns	ns	ns
FLT1	ns	ns	ns	ns	ns	ns	0.017	+1.41	ns	ns	ns	ns
FLT4	0.029 *	+1.36	ns	ns	ns	ns	0.018	+1.55	ns	ns	ns	ns
FST	ns	ns	ns	ns	ns	ns	0.026	−2.65	ns	ns	ns	ns
GALK1	ns	ns	ns	ns	ns	ns	0.035	+1.25	ns	ns	ns	ns
GHR	ns	ns	0.023	+1.54	ns	ns	ns	ns	ns	ns	ns	ns
GLUT1	ns	ns	ns	ns	ns	ns	0.024	+1.37	ns	ns	ns	ns
GLUT4	ns	ns	ns	ns	ns	ns	0.025	+1.30	ns	ns	ns	ns
GSK3b	ns	ns	ns	ns	ns	ns	0.023	+1.38	0.029 *	+1.41	ns	ns
HGF	ns	ns	0.006	+1.61	ns	ns	ns	ns	ns	ns	ns	ns
HSD11B1	ns	ns	ns	ns	ns	ns	0.013	+1.40	ns	ns	ns	ns
IGFBP5	ns	ns	ns	ns	ns	ns	0.027	+1.26	ns	ns	ns	ns
KDR	ns	ns	ns	ns	ns	ns	0.018	+1.32	ns	ns	ns	ns
MAT2A	ns	ns	0.023	+1.22	ns	ns	ns	ns	ns	ns	ns	ns
MAT2B	ns	ns	ns	ns	ns	ns	0.025	+1.25	ns	ns	ns	ns
MET	ns	ns	ns	ns	ns	ns	ns	ns	0.023 *	+1.54	0.026 *	−1.50
MSTN	ns	ns	0.029	+1.56	ns	ns	ns	ns	ns	ns	ns	ns
MYF5	ns	ns	0.035	+1.29	ns	ns	ns	ns	ns	ns	ns	ns
MYF6	ns	ns	ns	ns	0.043	−1.38	ns	ns	ns	ns	ns	ns
MYH2	ns	ns	ns	ns	ns	ns	0.044	+1.69	ns	ns	ns	ns
MyoD1	nd	nd	nd	nd	ns	ns	ns	ns	0.009	+2.03	ns	ns
Myogenin	ns	ns	ns	ns	ns	ns	ns	ns	0.003	+1.86	ns	ns
NCAPD2	ns	ns	0.032	+1.29	ns	ns	ns	ns	0.030 *	+1.43	ns	ns
Pax7	ns	ns	ns	ns	ns	ns	ns	ns	0.003	+1.84	ns	ns
PC	ns	ns	ns	ns	ns	ns	0.011	+1.59	ns	ns	ns	ns
PDGFA	ns	ns	ns	ns	0.047	−1.29	ns	ns	0.028 *	+1.48	ns	ns
PDPK1	ns	ns	0.013	+1.28	ns	ns	ns	ns	ns	ns	ns	ns
PFKM	ns	ns	0.008	+1.31	ns	ns	ns	ns	ns	ns	ns	ns
PIK3CA	ns	ns	ns	ns	ns	ns	ns	ns	0.018 *	+1.54	ns	ns
PIK3CD	ns	ns	ns	ns	ns	ns	0.041	+1.47	ns	ns	ns	ns
PIK3CG	ns	ns	ns	ns	ns	ns	0.022	+1.42	0.039 *	+1.60	ns	ns
PPARA	ns	ns	0.032	+1.28	ns	ns	0.023	+1.39	ns	ns	ns	ns
PPARD	ns	ns	ns	ns	0.013	−1.50	ns	ns	ns	ns	ns	ns
PPARGC1A	ns	ns	0.040	+1.46	ns	ns	ns	ns	ns	ns	ns	ns
PRKAA2	ns	ns	0.023	+1.37	ns	ns	ns	ns	ns	ns	ns	ns
SPP1	ns	ns	ns	ns	ns	ns	ns	ns	ns	ns	0.043 *	+4.34
VEGFB	0.025 *	+1.14	0.012	+1.12	ns	ns	ns	ns	ns	ns	ns	ns
VEGFC	ns	ns	ns	ns	0.032	−1.51	ns	ns	ns	ns	ns	ns

CON—Standard diet; MET—Standard diet supplemented with methylating micronutrients; FC—fold change; ns—not significant; nd—not detectable; dpc—days post conception; dpn—days post natum; ^1^ Positive FC: CON < MET; negative FC: CON > MET; * corresponding *q* > 0.30.

## Supplementary Materials

The following files are available online at www.mdpi.com/2072-6643/9/1/74/s1, Table S1: Postnatal carcass characteristics related to percent lean mass, back fat, pH, and meat color, Table S2: Gene symbols, accession numbers, primer sequences, and statistics (variance components mediated by diet and diet*sex) of selected genes; see Material and Methods section for details, Table S3: Comparison of Fluidigm and LightCycler 480 results for selected transcripts to verify Fluidigm data.

## Abbreviations

The following abbreviations are used in this manuscript:

B	Birth
CON	Standard diet
dpc	days post conception
dpn	days post natum
FC	Fold change
IN	Insemination
MET	Standard diet supplemented with methylating micronutrients
nd	not detected
ns	not significant
PCA	Principle component analysis
qPCR	Quantitative polymerase chain reaction
w	week
